# Diabetic Striate Syndrome: an uncommon complication of diabetes requiring further conceptual expansion

**DOI:** 10.3389/fendo.2025.1546919

**Published:** 2025-09-08

**Authors:** Ling Yu, Hua Gu, Wenli Hu, Lei Yang

**Affiliations:** ^1^ Department of Endocrinology, Beijing Chaoyang Hospital, Capital Medical University, Beijing, China; ^2^ Department of Radiology, Beijing Chaoyang Hospital, Capital Medical University, Beijing, China; ^3^ Department of Neurology, Beijing Chaoyang Hospital, Capital Medical University, Beijing, China

**Keywords:** diabetes striatopathy, cerebral infarction, cerebral small vessel disease, etiology, chorea

## Abstract

**Background:**

Diabetic Striate Syndrome (DSS) is a rare complication of diabetes, clinically characterized by chorea-like involuntary movements and contralateral basal ganglia abnormalities on brain CT and MRI. The identification of atypical DSS cases has broadened our understanding of this complex metabolic disorder.

**Objective:**

To investigate the clinical manifestations, imaging features, and potential pathogenesis of DSS to enhance clinical awareness and understanding of the condition

**Methods:**

We retrospectively analyzed clinical data from eight patients diagnosed with DSS who were hospitalized in the Department of Neurology at Beijing Chaoyang Hospital, affiliated with Capital Medical University, between January 2017 and July 2024. Their clinical presentations and imaging findings were reviewed, and both typical and atypical features, as well as potential pathogenic mechanisms, were discussed in the context of relevant literature.

**Results:**

Among the eight patients, five were female. All patients had a history of hypertension, and seven had a prior diagnosis of diabetes. Three patients experienced acute or subacute cerebral infarction. Seven patients presented with hemichorea, while one patient exhibited bilateral chorea. Random blood glucose levels at onset ranged from 7.9 to 27.7 mmol/L, and glycated hemoglobin (HbA1c) levels ranged from 8.7% to 15.5%. One patient tested positive for urinary ketones, and one developed symptoms following a rapid drop in blood glucose. Head CT scans revealed high-density lesions in the basal ganglia in four patients. MRI showed T1-weighted hyperintensity in the basal ganglia in seven patients, including one case with bilateral involvement. Hospital stays ranged from 6 to 14 days. All patients showed clinical improvement, with one achieving complete resolution of symptoms. In three cases, symptom improvement was achieved through blood glucose control alone.

**Conclusion:**

DSS represents a syndrome with an expanding clinical and imaging spectrum as research progresses. In this study, we propose a refined definition of the disorder and reaffirm the hypothesis that both basal ganglia ischemia and hyperglycemia synergistically contribute to the development and progression of DSS.

## 1 Introduction

In outpatient and emergency settings, clinicians occasionally encounter patients presenting with chorea-like or ballismus movements in the context of hyperglycemia. This condition was predominantly observed in elderly individuals with diabetes and poorly glycemic control. The primary clinical manifestation was unilateral involuntary limb movements. Neuroimaging studies (1, 2) typically revealed hyperdensity in the contralateral basal ganglia on brain CT and T1-weighted hyperintensity in the same region on brain Magnetic Resonance Imaging (MRI). Urinary ketone bodies are usually negative, and symptoms generally improve or resolve following appropriate glycemic control.

Historically, Diabetic Striate Syndrome (DSS) has been referred to by various names, including diabetic striatopathy (1–4), diabetic hemichorea/hemiballismus (5, 6), non-ketotic hyperglycemic hemichorea (7–10). As clinical awareness of this disorder has grown, an increasing number of atypical cases have been reported. These include patients with positive ketone bodies, those presenting with clinical symptoms in the absence of characteristic imaging findings, individuals showing imaging abnormalities without overt symptoms, cases of chorea-like movements following rapid glycemic correction, and patients with non-choreiform involuntary movements (2, 3, 7, 11). To better capture the heterogeneous clinical and radiological presentations, some scholars over the past 15 years have advocated for the use of the term diabetic striatopathy, reflecting a broader and more inclusive understanding of the disorder. However, given the clinical and radiological heterogeneity of this condition, we propose the term DSS as a more inclusive and accurate designation.

This paper summarizes both the typical and atypical manifestations of DSS observed in patients treated at our hospital over the past seven years, and reviews recent literature to provide an updated perspective on the syndrome.

## 2 Materials and methods

### 2.1 Study subjects and methods

This study included eight patients diagnosed with DSS admitted to the Department of Neurology at our hospital between January 2017 and July 2024. The inclusion criteria were as follows: (1) a previous diagnosis of diabetes or a new diagnosis of diabetes upon admission; (2) the presence of choreiform involuntary movements; (3) characteristic imaging abnormalities. Exclusion criteria included other known causes of chorea, such as Wilson’s disease, Huntington’s disease, severe hepatic or renal dysfunction leading to involuntary movements, Sydenham’s chorea, thyroid disorders, severe electrolyte imbalances, toxic exposures, infections, and autoimmune diseases.

We collected general demographic information, clinical data, laboratory test results, and neuroimaging findings for each patient. Laboratory and imaging assessments included blood glucose, glycated hemoglobin (HbA1c), complete blood count, liver and kidney function tests, electrocardiogram (ECG), head computed tomography (CT), magnetic resonance imaging (MRI), and magnetic resonance angiography (MRA).

All imaging data were independently reviewed by a radiologist and a neurologist. The analysis focused on identifying lesion locations on CT and MRI, as well as detecting cerebrovascular abnormalities, such as arterial stenosis.

## 3 Results

### 3.1 General data

A total of eight patients were included in the study, five of whom were female (62.5%). The age of onset ranged from 50 to 88 years, with a mean age of 76.8 years. Seven patients had a pre-existing diagnosis of diabetes with a disease duration ranging from 6 to 20 years, while one patient was newly diagnosed upon admission. All patients had a history of hypertension. Additionally, four patients had a history of coronary artery disease, and three presented with renal function abnormalities. Two patients had acute cerebral infarction—one involving the ipsilateral basal ganglia and the other a lacunar infarction in the corpus callosum. Another patient had a subacute cerebral infarction.

Seven patients exhibited unilateral choreiform movements, while one patient presented with bilateral symptoms. The first patient had undergone a head MRI 45 days prior to the onset of chorea due to complaints of dizziness and fatigue; imaging revealed T1-weighted hyperintensity in the left basal ganglia. Choreiform movements developed after a rapid reduction in blood glucose levels. Similarly, the second patient experienced dizziness preceding the onset of chorea and had positive MRI findings. This patient also experienced a recurrence of symptoms one month after the initial episode.

Neurological examination revealed decreased muscle tone in four patients, positive pathological reflexes on the symptomatic side in two patients, and mildly reduced muscle strength in one patient. Further clinical details are provided in **Table 1**.

**Table 1:** Clinical characteristics of 8 cases with Diabetic Striate Syndrome.

Case	Sex	Age (years)	Type of DM	Age of diabetes diagnosis	FBG on admission (mmol/l)	HbA1c levels (%)	Current medications	FBG before discharge (mmol/l)	Diabetes complications
1	F	74	2	67	8.6	14	Lixisenatide, Humalog	8.3	DPN, DR
2	F	82	2	62	8.2	8.7	Pioglitazone, Actos	7.1	DPN, DR, DN
3	M	50	2	44	11.9	15.5	NovoRapid, Levemir	5.3	DPN, DR, ACI
4	F	88	2	72	19.5	14.6	NovoLog, Levemir.	7.7	DPN, DR
5	F	81	2	58	6.7	11.2	Glucotrol, Acarbose.	5.3	DPN, DR, DN, ACI
6	M	69	2	49	6.3	11.3	NovoMix 30R, Metformin	6	DPN, DR, DF
7	M	86	2	85	5	9	Repaglinide	4.4	DPN
8	F	84	2	74	8.5	12.1	Acarbose, Glipizide	7.2	DPN

DM, Diabetes Mellitus; FBG, fasting blood glucose; F, female; M, male; DPN, diabetic peripheral neuropathy; DN, diabetic nephropathy; DR, diabetic retinopathy; DF, diabetic foot; ACI, acute cerebral infarction.

### 3.2 Laboratory tests

At the time of symptom onset, random blood glucose levels among the eight patients ranged from 7.9 to 27.7 mmol/L. Fasting blood glucose levels ranged from 5.0 to 19.5 mmol/L, with a median value of 8.35 mmol/L. Glycated hemoglobin (HbA1c) levels ranged from 8.7% to 15.5%, with a median of 11.7%. Serum osmolality values were between 280 and 300 mOsm/L. One patient tested positive for urinary ketones. Detailed laboratory data are presented in **Table 1**.

### 3.3 Imaging findings

**Figure 1:**
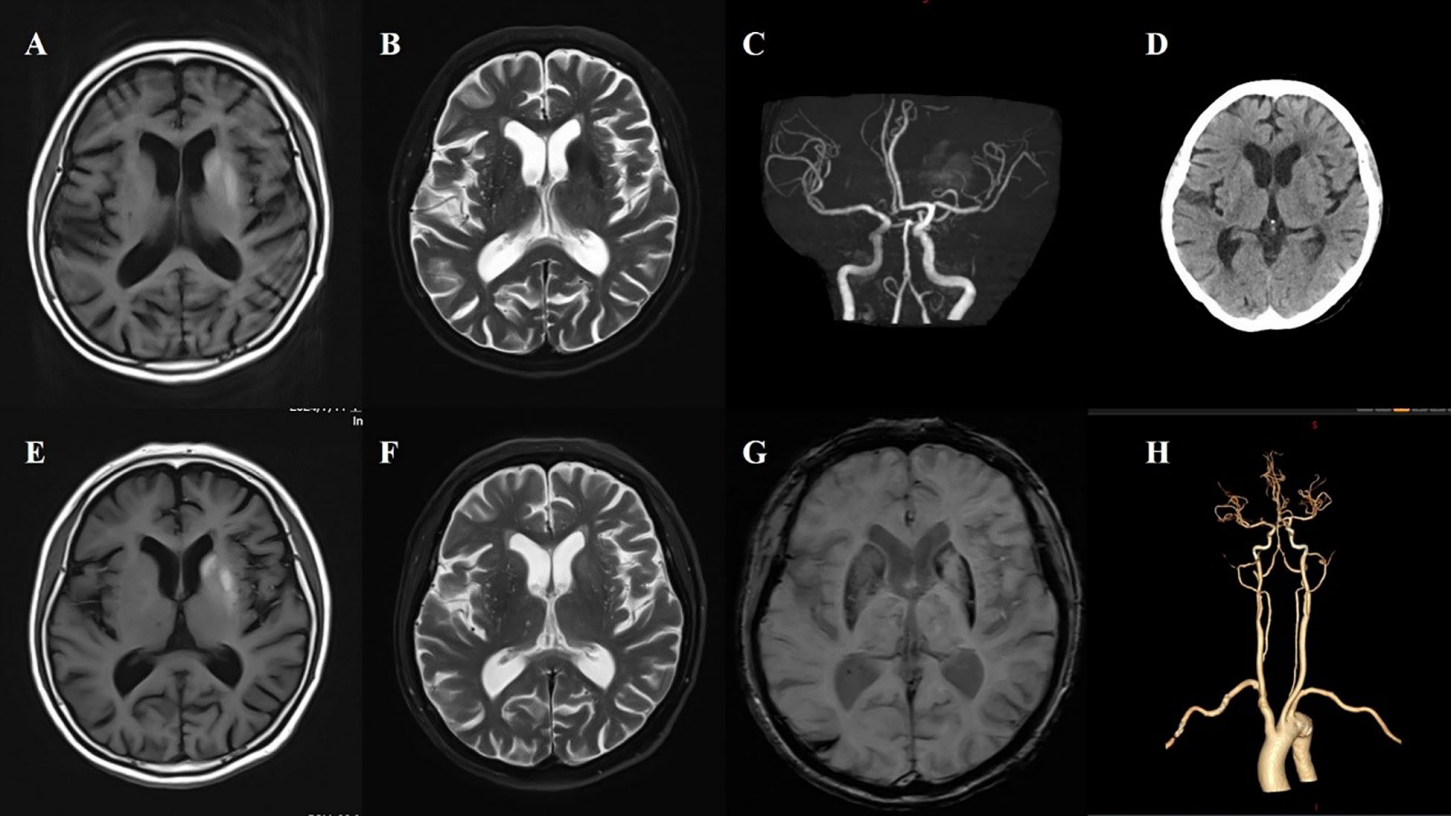
Case 1. **(A)** The first MR T1 image demonstrated hyperintensity in the left putamen and the head of the caudate. **(B)** The first MR T2 image revealed hypointensity in the left putamen and the head of the caudate. **(C)** Brain MRA indicated mild-moderate stenosis of left MCA. **(D)** Brain CT showed no significant abnormalities in basal ganglia. **(E)** A follow-up brain MR T1 image after one month showed high signals were more pronounced in the left putamen and the head of the caudate. **(F)** The second brain MR T2 image revealed that the lesion on the left side changed from a low signal to a high signal and isointense. **(G)** SWI sequence shows asymmetric low signals in the bilateral basal ganglia (putamen and the head of the caudate), with the left side being more pronounced. **(H)** CTA indicated mild-moderate stenosis of left MCA and multiple intracranial vascular stenosis.

**Figure 2:**
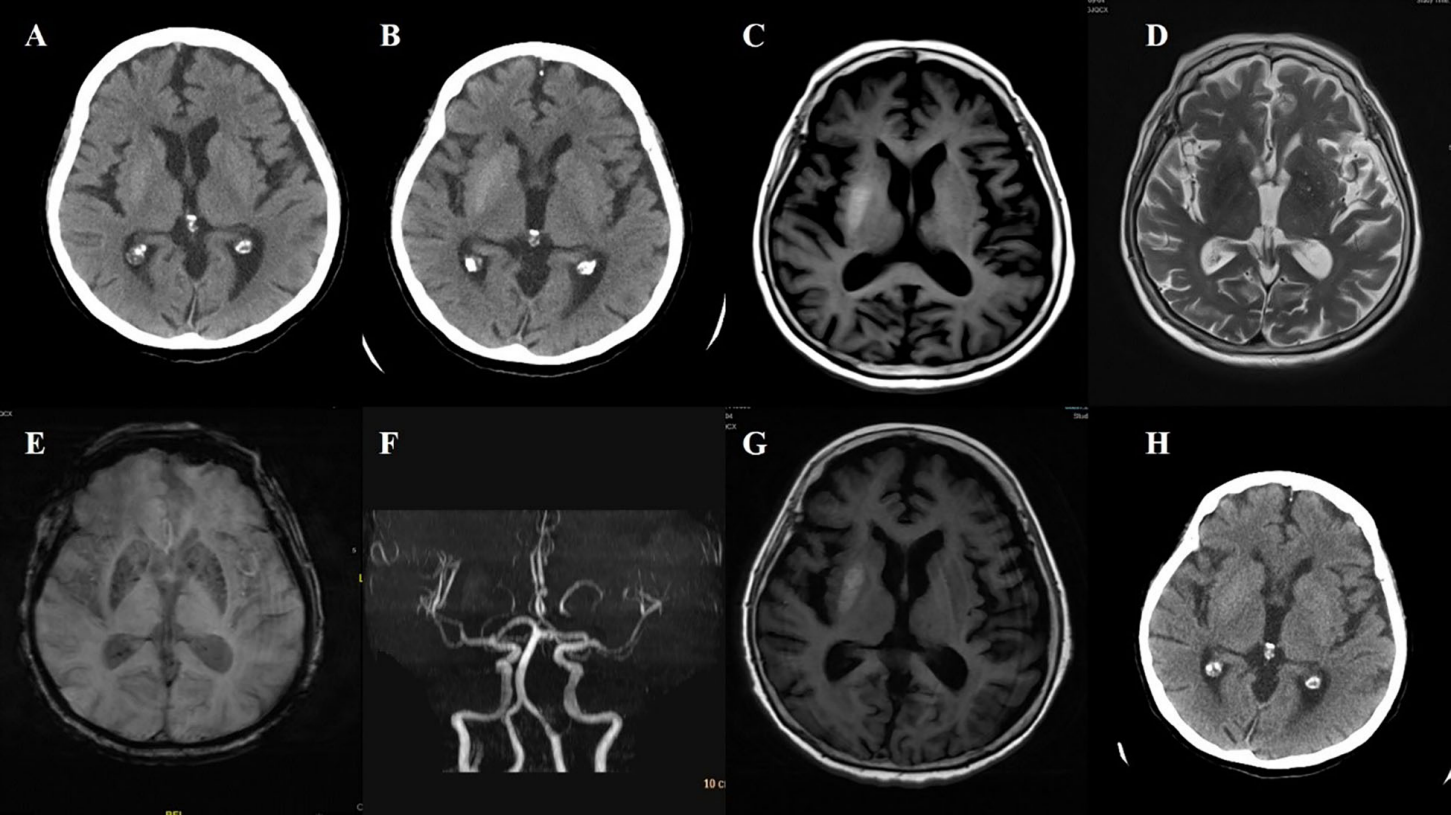
Case 2. **(A)** The initial Brain CT scan demonstrated a slightly higher density in the right putamen. **(B)** The second brain CT scan showed an increase in density in the right putamen compared to the first. **(C)** Brain MR T1 image demonstrated hyperintensity in the right putamen. **(D)** Brain MR T2 image revealed hypointensity in the right putamen. **(E)** The SWI sequence showed scattered slightly low signals in the bilateral basal ganglia. Compared to the left side, no significant low signals were observed in the right putamen. **(F)** Brain MRA revealed atherosclerosis of the intracranial arteries, with poor visualization of the distal middle cerebral artery. **(G)** During the second hospitalization, brain T1 image showed a decreased hyperintensity of the right putamen. **(H)** Brain CT also found that the high density in the right putamen has decreased compared to the first one.

**Figure 3:**
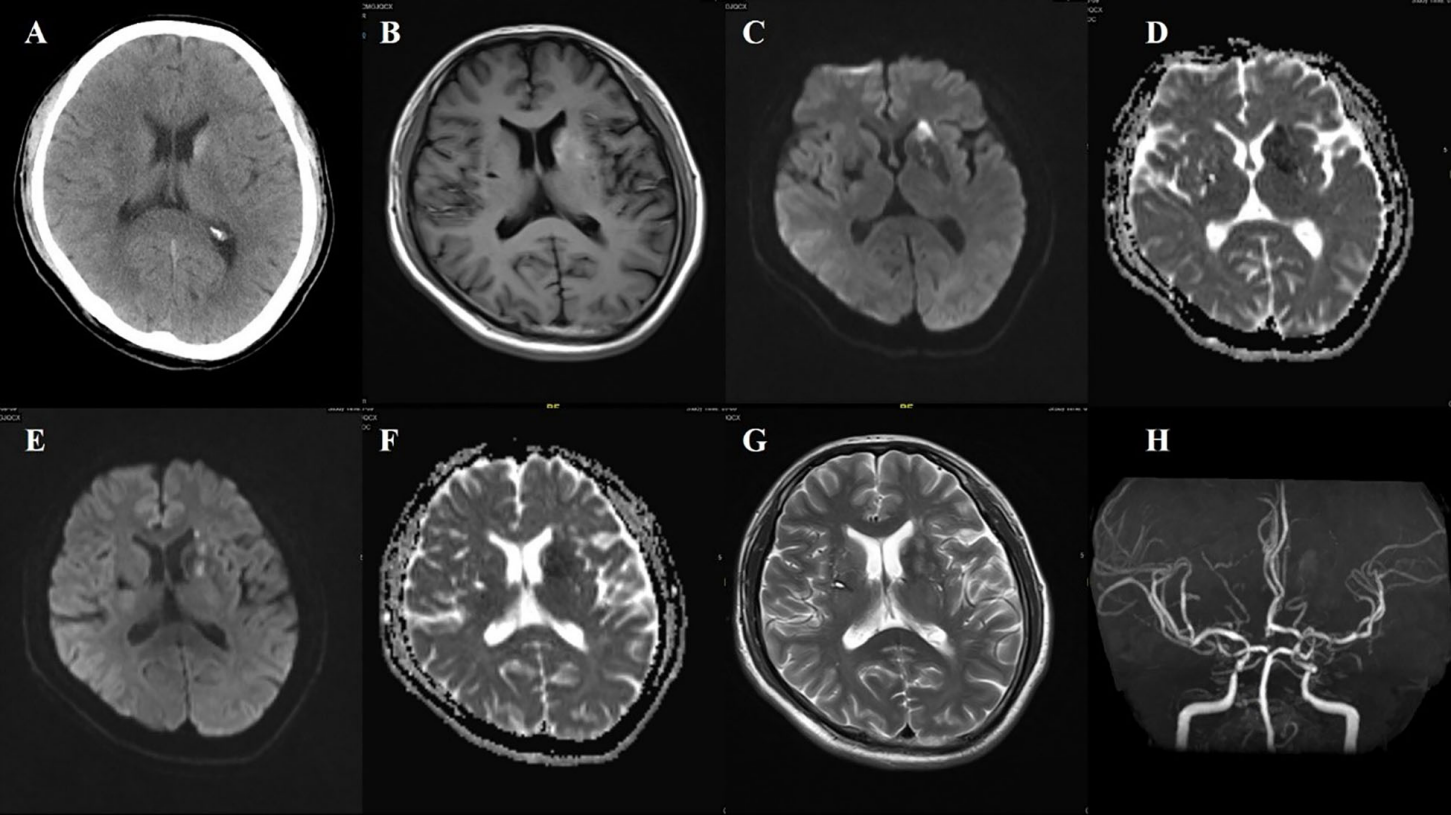
Case 3. **(A)** Brain CT revealed a slightly higher density in the left head of the caudate. **(B)** Brain MR T1 image demonstrated hyperintensity in the left putamen and the head of the caudate. DWI sequence shows scattered high signals in the head of the left caudate nucleus **(C)** and the anterior limb of the internal capsule **(E)**. The corresponding caudate nucleus **(D)** and the anterior limb of the internal capsule **(F)** show low signals on the ADC map. **(G)** MR T2 sequence showed abnormal high signals in the non-DWI high signal areas of the head of the caudate nucleus, and other scattered high signal areas of acute infarction were also slightly hyperintense. **(H)** Brain MRA indicated mild-moderate stenosis of left MCA and multiple intracranial vascular stenosis.

**Figure 4:**
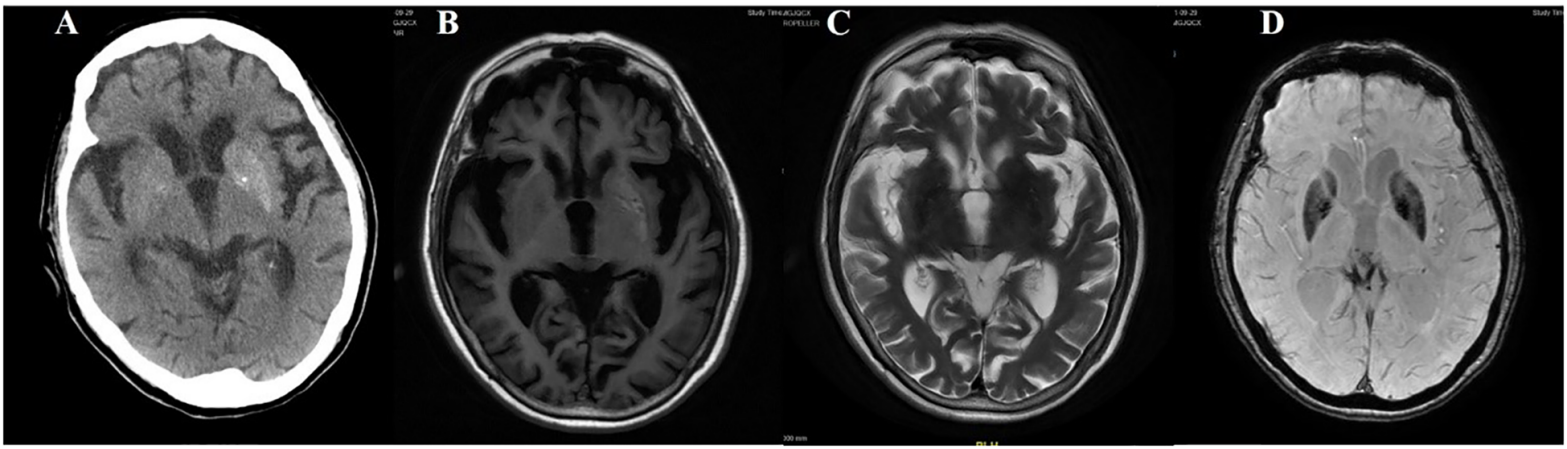
Case 4. **(A)** Brain CT scan demonstrated a slightly higher density in the left putamen. **(B)** Brain MR T1 image demonstrated hyperintensity in the left putamen. **(C)** Brain MR T2 image revealed hypointensity in the left putamen. **(D)** The SWI sequence showed scattered slightly low signals in the bilateral basal ganglia, more prominent on the left side.

**Figure 5:**
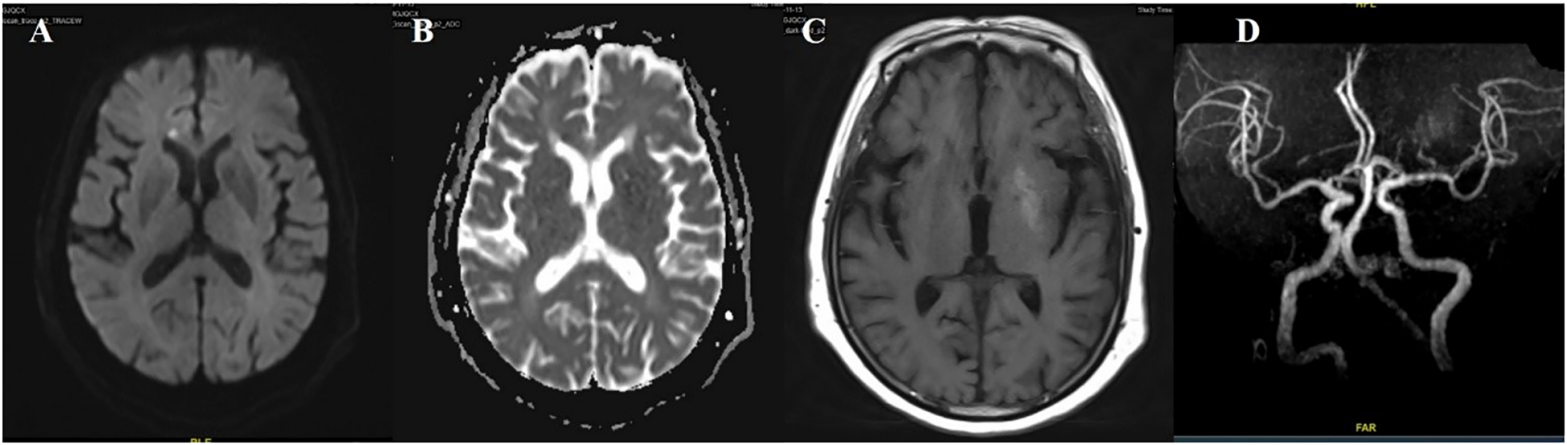
Case 5. DWI sequence shows high signals in corpus callosum **(A)**. The corresponding area **(B)** show low signals on the ADC map. **(C)** Brain MR T1 image demonstrated hyperintensity in the left putamen. **(D)** Brain MRA indicated mild-moderate stenosis of left MCA. .

**Figure 6:**
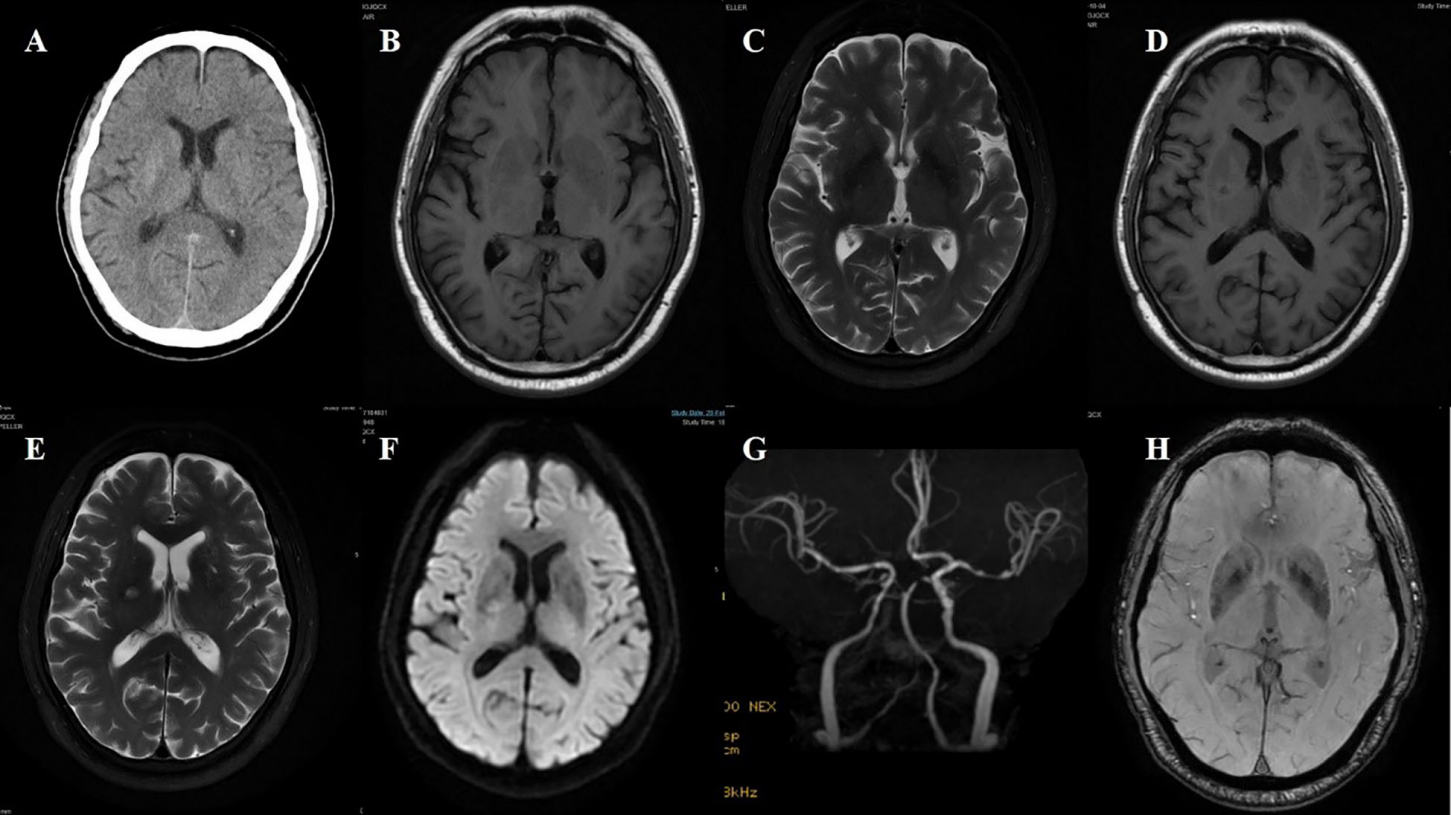
Case 6. **(A)** Brain CT revealed a slightly higher density in the right putamen. Brain MR T1 image **(B)** and T2 image **(C)** demonstrated no significant abnormal signals. On the adjacent slice, the right putamen showed a slightly hyperintense signal with a central hypointense signal on the T1 sequence **(D)**, and a slightly hyperintense punctate signal on both the T2 **(E)** and DWI **(F)** sequences. **(G)** Brain MRA indicated moderate-severe stenosis of bilateral MCA and multiple intracranial vascular stenosis. **(H)** The SWI sequence showed scattered asymmetric slightly low signals in the bilateral basal ganglia.

**Figure 7:**
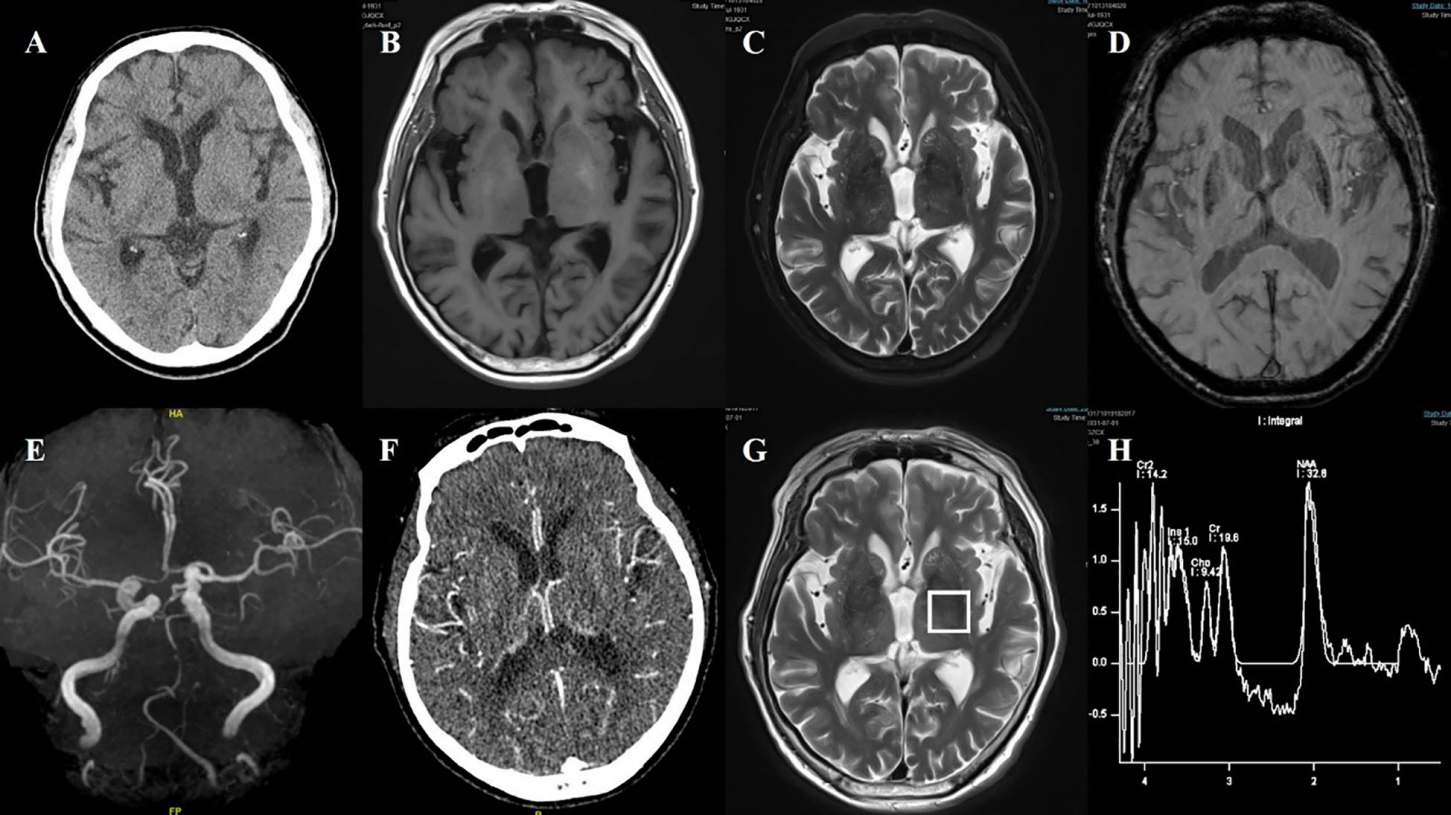
Case 7. **(A)** Brain CT showed no significant abnormalities in basal ganglia. **(B)** Brain MR T1 image revealed hyperintensity in the bilateral putamen. **(C)** Brain MR T2 image revealed hypointensity in the bilateral putamen. **(D)** The SWI sequence showed scattered asymmetric slightly low signals in the bilateral putamen and the head of the caudate. **(E)** Brain MRA revealed atherosclerosis of the intracranial arteries, with poor visualization of the posterior circulation system. **(F)** Brain CT perfusion raw images suggested that the bilateral vessels were essentially symmetric. **(G, H)** Brain MRI spectroscopy did not reveal abnormalities in the bilateral basal ganglia region.

**Figure 8:**
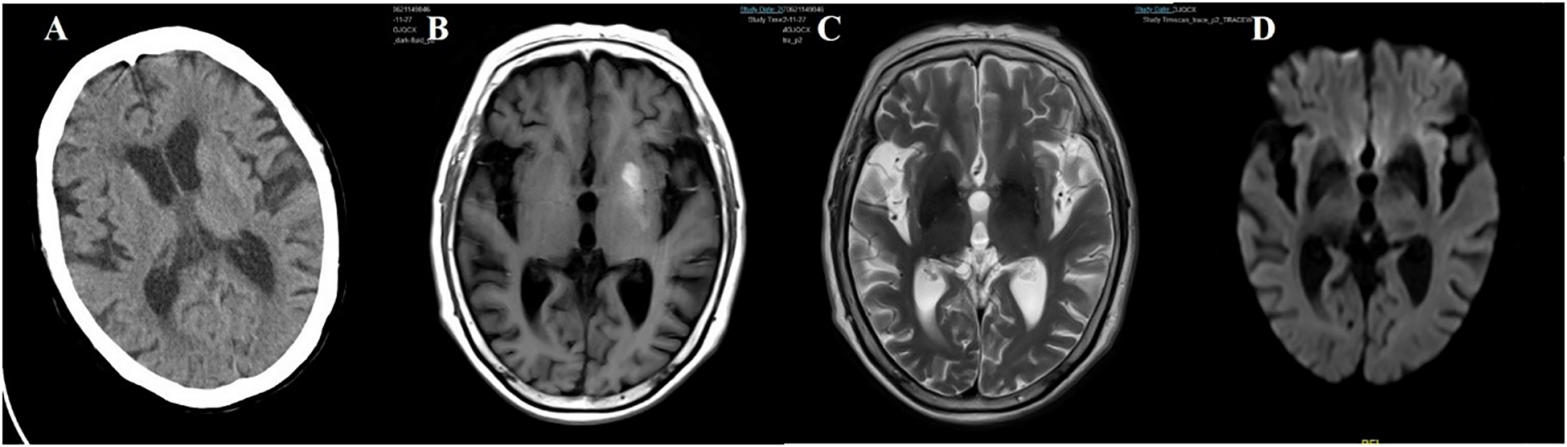
Case 8. **(A)** Brain CT scan demonstrated a slightly higher density in the left putamen. **(B)** Brain MR T1 image demonstrated hyperintensity in the left putamen. **(C)** Brain MR T2 image revealed hypointensity in the bilateral putamen. **(D)** The DWI sequence showed low signals in the bilateral basal ganglia.

Routine head CT revealed abnormalities in the basal ganglia in four patients, one of whom did not exhibit corresponding lesions on T1-weighted MRI. Among the remaining seven patients, one demonstrated bilateral lesions on MRI. In two patients, the lesions involved both the caudate nucleus and the putamen, whereas in the other six patients, the lesions were confined to the putamen. On T2-weighted imaging, five patients showed slightly hypointense signals, two exhibited isointense signals, and one presented with slightly hyperintense signals. Diffusion-weighted imaging (DWI) revealed slightly hypointense or hypointense signals in six patients, while the remaining two had isointense signals. On susceptibility-weighted imaging (SWI), seven patients exhibited slightly hypointense signals in the affected basal ganglia regions, and one showed isointense signals. Six patients underwent magnetic resonance angiography (MRA), among whom five demonstrated varying degrees of stenosis in the middle cerebral artery (MCA) on the side corresponding to the lesion. Further details are presented in **Table 2** and **Figures 1**–**8**.

**Table 2:** Imaging characteristics of 8 cases with Diabetic Striate Syndrome.

Case	Brain CT	Location of lesion	MRI				MCA stenosis	Additional medication	Hospitalization duration (days)	Course
			T1	T2	DWI	SWI				
1	Normal	CN, PN	H	SH	SL	SL	Y	Y	7	Improved
2	P	PN	H	SL	SL	SL	Y,B	Y	14	Improved
3	P	CN, PN	H	SL	SL	I	Y	N	12	Improved
4	P	PN	H	SL	SL	SL	/	Y	13	Improved
5	Normal	PN	H	I	I	SL	Y	Y	6	Improved
6	P	PN	N	I	I	SL	Y	N	7	Relieved
7	Normal	PN	H	SL	L	SL	Normal	Y	13	Improved
8	P	PN	H	SL	L	SL	/	N	7	Improved

B, bilateral; CN, caudate nucleus; I, isointense signal; N, negative; P, positive; PN, putamen nucleus; H, high signal; SH, slightly high signal; L, low signal; SL, slightly low signal; Y, yes; N, no,/, not done.

### 3.4 Treatment and outcomes

All eight patients received glycemic control as part of their treatment. Three patients were managed with glycemic control alone, while the remaining five received additional symptomatic therapy aimed at alleviating choreiform movements. Blood glucose management strategies were developed in consultation with endocrinologists. Patients in the table were listed in descending order of age. Notably, a shift in treatment patterns can be observed over time, with the medications used in more recent years differing from those administered in earlier cases. The duration of hospitalization ranged from 6 to 14 days. All patients experienced symptom improvement, with one patient achieving complete remission.

## 4 Special case reports

### 4.1 Case 1

A 74-year-old female patient was admitted with a chief complaint of “involuntary movement of the right lower limb for one week.” The symptoms were characterized by purposeless, uncontrollable twisting movements of the right lower limb, primarily involving flexion-extension at the proximal joints and rotational movements at the foot and ankle. The movements were continuous during wakefulness and subsided during sleep. Emergency brain CT showed no significant abnormalities. The patient’s fasting blood glucose on admission was 8.6 mmol/L.

Her past medical history included a 7-year history of diabetes, a 2-month history of hypertension (untreated), and recent intermuscular venous thrombosis of the lower limbs diagnosed one month prior. One month before admission, her random blood glucose level was 30 mmol/L, and her glycated hemoglobin (HbA1c) was 14%. She was initiated on insulin glargine, liraglutide, insulin lispro (Humalog), dapagliflozin, and pioglitazone. During this treatment, her blood glucose dropped to between 3.8 and 4.1 mmol/L.

Review of the patient’s medical history revealed that 45 days before hospitalization, she experienced dizziness and fatigue and underwent brain MRI in the neurology department. The MRI revealed hyperintensity in the left basal ganglia on T1-weighted imaging, slight hypointensity on T2-weighted and fluid-attenuated inversion recovery (FLAIR) sequences, and multiple enlarged perivascular spaces (EPVS) in the bilateral basal ganglia region on T2-weighted imaging. At that time, she did not present with involuntary movements. Neurological examination upon admission showed decreased muscle tone in the right limb, with no other significant abnormalities.

Laboratory tests after admission revealed a fasting blood glucose of 8.6 mmol/L and negative urine ketone bodies. Liver and kidney function tests, complete blood count, and electrolytes were within normal limits. A repeat brain MRI showed persistent hyperintensity in the left basal ganglia on the T1-weighted sequence, more pronounced than before, with slightly increased signal on the T2-weighted sequence and slight hypointensity on susceptibility-weighted imaging (SWI). Brain computed tomography angiography (CTA) demonstrated stenosis of the M1 segment of the left middle cerebral artery (MCA) and multiple sites of intracranial arterial stenosis. Following admission, the patient’s symptoms gradually improved with the addition of haloperidol (**Figure 1**).

### 4.2 Case 3

A 50-year-old male patient was admitted with a chief complaint of “involuntary movement of the right limbs for one week.” The symptoms were characterized by purposeless, uncontrollable movements involving both the right upper and lower limbs. The upper limb displayed flexion-extension of the arm, flinging movements, and wrist rotation. The lower limb showed proximal flexion-extension and distal foot and ankle rotation. The movements were continuous during wakefulness but diminished significantly or disappeared during sleep. Emergency brain CT revealed slightly increased density in the head of the left caudate nucleus, raising suspicion for hemorrhage, calcification, or abnormal substance deposition, along with multiple lacunar infarcts. The patient’s random blood glucose was 27.7 mmol/L, and urine ketone bodies were positive at 10 (+) mg/dl.

The patient had a 6-year history of hypertension, which was untreated, and a 6-year history of diabetes. He had previously taken glimepiride, although the dosage was unknown, and had discontinued the medication three months prior. He denied any history of stroke or coronary artery disease.

Neurological examination revealed decreased muscle tone in the right limbs, with no other notable findings. After admission, repeat random blood glucose was 33.8 mmol/L, HbA1c was 15.5%, urine glucose was (++++), and urine ketone bodies turned negative. Liver and kidney function tests, complete blood count, electrolytes, and thyroid function were within normal limits. On the third day of hospitalization, brain MRI revealed abnormal signals in the left basal ganglia, with findings on the T1-weighted sequence suggestive of possible acute infarction or hemorrhage. However, the lesion was not located in the caudate nucleus head. T2-weighted imaging showed scattered and relatively prominent enlarged perivascular spaces (EPVS) in the left basal ganglia region. Magnetic resonance angiography (MRA) indicated moderate to severe stenosis in the bilateral internal carotid arteries (C2–C6 segments), stenosis of the left middle cerebral artery (M1 segment), and multiple stenoses in the bilateral posterior cerebral arteries (P1–P2 segments). Susceptibility-weighted imaging (SWI) did not reveal hemorrhagic changes corresponding to the high-density lesion seen on CT in the caudate nucleus (**Figure 3**).

The patient received antiplatelet and lipid-lowering therapy. After five days of treatment, his fasting blood glucose decreased to 7.8 mmol/L, and 2-hour postprandial glucose levels ranged from 9.5 to 12.8 mmol/L. The involuntary movements improved significantly.

## 5 Discussion

DSS also known as diabetic striatopathy, diabetic non-ketotic hemichorea/hemiballism, was characterized by hyperglycemia associated with both chorea/ballism and contralateral striatal lesion. In 1960, Bedwell first reported this rare complication of diabetes (7, 11). Current research suggests that the incidence of this condition is less than 1 in 100,000 (3). However, differing understandings of the condition among scholars may mean that this figure underestimates its true incidence.

The literature on this condition predominantly consists of retrospective case reports, which often adhere to the classical definition of DSS-namely, non-ketotic hyperglycemia, chorea, and contralateral basal ganglia hyperdensity on CT and/or T1-weighted hyperintensity on MRI. The lesions typically have well-defined borders and lack surrounding edema. The vast majority of case reports have used this classical definition of DSS, which involves the presence of involuntary movements and striatal abnormalities on imaging in patients with type 2 diabetes mellitus. Studies indicate that some patients exhibit involuntary movements without corresponding radiographic evidence, and such cases may not have been included in clinical reports. Because of the varying definitions of DSS among researchers, the epidemiological data for this condition remain inconsistent.

Over a decade ago, scholars proposed five subtypes of DSS (12), including: the classical type; a type with symptoms and imaging changes but without hyperglycemia; non-ketotic hyperglycemia with symptoms but no imaging changes; non-ketotic hyperglycemia with imaging changes but no symptoms; and a type with non-ketotic hyperglycemia accompanied by bilateral symptoms and imaging changes.

Among the six cases we presented, several exhibited non-classic features. Some patients had radiographic lesions without corresponding symptoms (13), while in case 1, chorea-like symptoms appeared following rapid glycemic control (14). There have also been reports of patients with diabetes exhibiting typical DSS manifestations during hypoglycemic episodes. One possibility was that the striatum, being highly sensitive to changes in blood glucose levels, is particularly vulnerable to damage. The literature notes that chorea-like symptoms can persist for extended periods in some patients, however, recurrent episodes of chorea are rarely reported (15). Our second patient experienced a recurrence of chorea-like symptoms one month after the initial episode. Co-occurrence with acute cerebral infarction has been previously reported (8, 9, 16–19). Our third, fifth and sixth patients had recent infarctions. Notably, the third patient had an acute cerebral infarction on the same side as the abnormal basal ganglia lesion, which has been rarely reported (16, 18). Furthermore, this patient’s initial urinalysis showed positive ketone bodies, which does not align with the classical DSS presentation. Clinically, patients presenting with bilateral chorea have also been observed (20, 21), as in our seventh patient. Interestingly, some patients with bilateral striatal lesions may exhibit only unilateral choreiform symptoms.

As more atypical cases have been reported, the definition of DSS warrants further expansion. Some scholars have proposed a broader definition of the condition (3), characterizing it as a hyperglycemic state associated with at least one of the following: (1) chorea or ballism; (2) striatal hyperdensity on CT or hyperintensity on T1-weighted MRI. This definition already encompasses the majority of patients seen in clinical practice. However, there were still some patients who develop chorea-like symptoms after their blood glucose levels were lowered (14, 22). Additionally, individuals with diabetes may exhibit other types of involuntary movements, including hemifacial spasm, dystonia of the limbs, tremor, and dystonic disorders (23–25).

We propose a slight revision to the current definition of DSS, patients with diabetes presenting with either of the following: (1) involuntary movement; (2) striatal hyperdensity on CT or hyperintensity on T1-weighted MRI. Furthermore, some researchers have suggested a classification system for diabetic striatopathy, dividing it into three subtypes (2). However, given the rarity of this condition in clinical settings, our team believes that further subclassification may not be conducive to clinical research or practical diagnosis.

Currently, the most widely accepted theory regarding the pathogenesis of DSS was that metabolic disturbances in the striatum, caused by hyperglycemia. According to the metabolic abnormality theory, a reduction in γ-aminobutyric acid (GABA) levels leads to dysfunction of both the direct and indirect basal ganglia pathways, resulting in decreased inhibition of the thalamus. This disinhibition enhanced thalamo-cortical activity, ultimately producing chorea-like movements (3, 7, 11).

The emergence of atypical cases has prompted further discussion among scholars. Pathological studies have confirmed ischemia-like infarct changes in the striatal region (4, 26), suggesting that ischemia may also play a role in the pathogenesis of DSS. In our cases 3, 5, and 6, patients had recent cerebral infarctions, while in cases 1, 3, 5, 6, and 7, there was evidence of responsible vascular lesions or intracranial arterial stenosis. Additionally, enlarged perivascular spaces (EPVS) in the basal ganglia were observed in cases 1, 2, 3, 6, and 7. Currently, DSS is believed to occur predominantly in elderly patients with poorly controlled diabetes—a population also at high risk for ischemic cerebrovascular disease. Our patients exhibited EPVS, which are biomarkers of cerebral small vessel disease, as well as evidence of intracranial arterial stenosis, further supporting the potential role of cerebrovascular pathology in the development of DSS.

Based on previous research (8, 9, 16–19) and our case findings, we hypothesized that ischemic mechanisms contribute to the onset and progression of DSS. First, pathological evidence from earlier studies supports the presence of infarct-like changes in the basal ganglia region (4, 26, 27). Second, the basal ganglia are supplied by deep penetrating lenticulostriate arteries, which lack collateral circulation, rendering this region particularly susceptible to ischemia. In our cohort, most patients exhibited intracranial arterial stenosis, especially involving the middle cerebral artery. Finally, our patients commonly presented with risk factors for cerebrovascular disease, along with imaging evidence of small vessel disease in the basal ganglia. Some patients also had concurrent acute or subacute cerebral infarction.

Previous studies evaluating cerebrovascular conditions in DSS patients have been limited (7), and to date, no reports have specifically assessed markers of cerebral small vessel disease such as EPVS. In our series, six patients underwent cranial MRA, with five showing middle cerebral artery stenosis. Additionally, most patients demonstrated EPVS in the basal ganglia region, with two cases classified as severe. These findings suggest that future evaluations of DSS patients should include assessment for both cerebral small vessel disease and large-vessel pathology.

Research on imaging findings in patients with DSS remains inconclusive. Various mechanisms have been proposed to explain the observed abnormalities, including petechial hemorrhage, infarction, myelinolysis, and calcium deposition (1, 11). Some pathological studies have also suggested a correlation between microbleeds and the characteristic imaging changes seen in DSS. Three studies (4, 26, 27) reported evidence of infarction in the basal ganglia region, two of them (4, 26) additionally identifying microbleeds and erythrocyte extravasation in the infarcted areas. In a study by Mestre et al., microbleeds were observed in the striatum, and the authors attributed the erythrocyte extravasation to blood-brain barrier dysfunction induced by hyperglycemia (10).

However, the findings of a study utilizing the T2* sequence did not support the microbleeding hypothesis, as the T2* signal changes did not correspond to imaging features typically seen in focal hemorrhage (28). Six patients with clinically documented hyperglycemia-induced hemichorea had undergone MR imaging with a T2*-based sequence. Among them, four exhibited unilateral hypointense signals within the striatum. Although SWI is currently considered highly sensitive for detecting microbleeds, none of our patients demonstrated microbleed-related signals on SWI sequences.

Based on previous literature and the characteristics observed in our case series, we believe that ischemia in the basal ganglia and hyperglycemia jointly contribute to the onset and progression of the disease.

## 6 Conclusion

As research advances, the clinical spectrum of diabetic striatopathy (DSS) continues to expand. We propose a revised definition of the syndrome and reaffirm that both basal ganglia ischemia and hyperglycemia play synergistic roles in its pathogenesis.

## Data Availability

The original contributions presented in the study are included in the article/supplementary material. Further inquiries can be directed to the corresponding author.
